# Relationship between radiosensitivity of peripheral blood lymphocytes, breast cancer cells, and breast cancer stem cells in patients with breast cancer

**DOI:** 10.1016/j.bbrep.2026.102733

**Published:** 2026-08-04

**Authors:** Mohammad Taghi Bahreyni Toossi, Hosein Azimian, Roham Salek, Seyed Abbas Tabatabaei, Mohammad Naser Forghani, Elham Dolat

**Affiliations:** a Medical Physics Research Center, Basic Sciences Research Institute, Mashhad University of Medical Sciences, Mashhad, Iran; b Cancer Research Center, Faculty of Medicine, Mashhad University of Medical Sciences, Iran; c Department of Clinical Pathology, Ghaem Hospital, Mashhad, Iran; d General Surgery Department, Omid Hospital, Mashhad, Iran; e Department of Medical Physics, Faculty of Medicine, Mashhad University of Medical Sciences, Mashhad, Iran

**Keywords:** Breast cancer, Personalized radiotherapy, Cancer stem cells, SF2, γH2AX

## Abstract

Radiosensitivity (RS) is an important biological factor that may contribute to future radiotherapy individualization. However, a comprehensive metric that adequately captures the complexities of radiation sensitivity has not yet been proposed, and the survival fraction at 2 Gy (SF2) remains the prevailing gold standard. Considerable variability in radiation responses across different cell types, together with heterogeneity within neoplastic and normal cell populations, continues to complicate radiosensitivity assessment.

This study aimed to investigate the relationship between the radiosensitivity of malignant and non-malignant cells, with particular emphasis on cancer stem cells (CSCs). Blood samples and tumor biopsy specimens were collected from 20 breast cancer patients. Radiosensitivity was evaluated using clonogenic survival assays in lymphocytes, breast cancer cells, and breast cancer stem cells, while DNA damage and repair kinetics in lymphocytes were assessed using the γH2AX assay.

The results showed that the geometric mean measured 30 min after radiation exposure, as well as the residual double-strand breaks (DSBs) at 3 and 24 h in the γH2AX assay, were significantly correlated with lymphocyte SF2. These findings suggest that the γH2AX assay may provide complementary information to clonogenic survival assay.

However, no significant correlation was observed between the radiosensitivity of lymphocytes and that of tumor cells or CSCs. Breast cancer stem cells exhibited slightly higher survival following irradiation compared with the corresponding tumor cells, indicating a trend toward increased radioresistance.

The observed radiosensitivity patterns may provide preliminary biological insights, but they should not be interpreted as direct clinical predictors without validation in larger cohorts with treatment outcome data. Further studies incorporating larger cohorts and additional biological indicators are required to improve the prediction of radiation response and clarify their potential relevance for future individualized radiotherapy research.

## Highlighted

• Radiosensitivity is essential for selecting patients for personalized RT.
• Radiation response differs markedly across cell and tumor/normal populations.
• Residual γH2AX DSB at 3–24 h shows strong correlation with lymphocyte SF2.
• γH2AX residuals may provide a practical alternative to clonogenic testing.
• Tumor and CSC radiosensitivity show no link to lymphocyte-based indices.

## Graphical abstract

**Figure:**
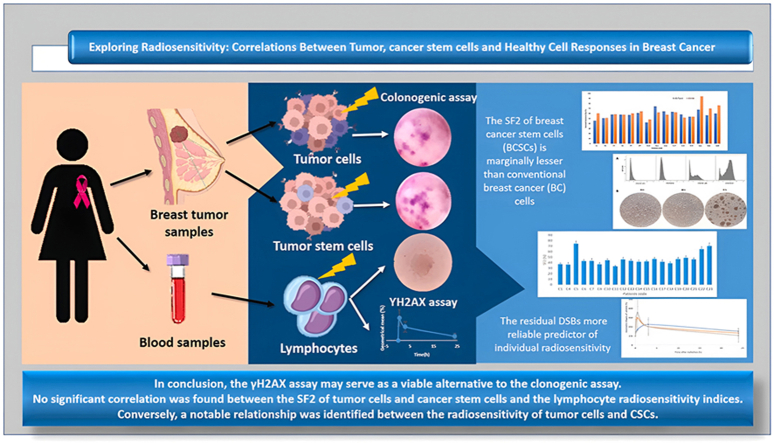

## 1 Introduction

Personalized medicine has increasingly shaped contemporary oncology by adapting therapeutic approaches to the molecular and clinical characteristics of individual patients [1]. This concept is particularly significant in breast cancer (BC), which remains the most frequently diagnosed malignancy among women worldwide. In 2020 alone, approximately 2.3 million cases were reported, reflecting the substantial global burden of this disease [2]. The heterogeneous nature of BC, along with its diverse histopathological features, highlights the need for individualized therapeutic strategies to optimize outcomes [3]. Current guidelines recommend adjuvant systemic therapy and radiotherapy following breast-conserving surgery or mastectomy. Endocrine therapy is offered to patients with hormone receptor–positive disease, while those with HER2-positive tumors receive targeted anti-HER2 agents. In early-stage BC, several multigene assays are used as prognostic tools to estimate recurrence risk [4,5].

Radiotherapy (RT) is essential in the management of breast cancer. It uses ionizing radiation to eradicate malignant cells to while sparing surrounding healthy tissues as much as possible [[6], [7], [8]]. Over the past decade, the development of advanced techniques, including intensity-modulated radiation (IMRT), volumetric modulated arc therapy (VMAT), tomotherapy, and hadron therapy, has improved dose distribution and reduced toxicity, especially in cases requiring regional nodal irradiation or in patients with challenging breast anatomy [[9], [10], [11]].

In parallel with technological advances, artificial intelligence has been introduced into various stages of oncologic imaging, with the goal of improving tumor characterization and clinical decision-making. Despite promising early results, the widespread adoption of AI-based tools in routine practice remains limited [[12], [13], [14], [15], [16]]. Beyond imaging, genomic profiling has become an established method for stratifying patients, particularly through assessment of HER2 status or expression-based signatures. Widely used gene panels such as Oncotype DX and MammaPrint help estimate recurrence risk in early-stage disease [4,17]. However, the clinical utility and appropriate indications for these tests are still debated, and many clinicians apply them selectively [[18], [19], [20]].

Despite advances in personalized approaches, including genome-based models for RT dose modulation [21] and radiosensitivity indices [22], challenges remain in the effective individualization of RT dosing based on tumor radiosensitivity. Although radiosensitivity indices show promise, their validation through larger prospective studies is critical [23,24].

Moreover, intratumoral heterogeneity complicates therapeutic response and contributes to recurrence, partly due to cancer stem cells that exhibit radioresistant behavior. These cells possess self-renewal and differentiation capacity, express ATP-binding cassette (ABC) transporters, and activate signaling pathways such as Wnt, Notch, and BRCA1/2, enabling their survival and potential for metastasis [[25], [26], [27], [28], [29], [30]].

Radiosensitivity remains a critical determinant of RT efficacy, yet conventional assays—particularly clonogenic survival assays—are labor-intensive and poorly suited for clinical use. Consequently, alternative biomarkers and measurement strategies have been an area of ongoing interest in radiobiology [31,32 33]. A deeper understanding of radiosensitivity variation among different cellular compartments may support more precise RT dose selection and improve therapeutic outcomes.

This study investigates the relationship between the radiosensitivity of peripheral blood lymphocytes, breast cancer cells, and Breast cancer stem cells in patients with breast cancer, providing preliminary data that may inform future research on individualized radiotherapy approaches.

## 2 Materials and methods

### 2.1 Subjects

Twenty women with histologically confirmed breast cancer were enrolled in this study. The mean age of the participants was 50 ± 9.23 years (range: 35–70 years). Patients were recruited prior to receiving any systemic therapy, including chemotherapy or radiotherapy, to avoid potential treatment-related alterations in radiosensitivity.

The study protocol was approved by the Ethics Committee of Mashhad University of Medical Sciences (approval code: IR.MUMS.MEDICAL.REC.1398.533), and written informed consent was obtained from all participants. All experimental procedures were conducted in accordance with relevant ethical guidelines and regulations.

Tumor samples were characterized using immunohistochemical analysis according to the 2020 ASCO/CAP guidelines for breast cancer biomarkers.

### 2.2 Blood sampling and isolation of peripheral blood mononuclear cells

Peripheral blood samples were collected in heparinized tubes and processed within a short time after collection. Peripheral blood mononuclear cells (PBMCs) were isolated using density-gradient centrifugation with Ficoll (Innotrain, Germany) according to the manufacturer's instructions.

Following centrifugation, PBMCs were washed twice with phosphate-buffered saline (PBS, Sigma) to remove residual plasma and Ficoll. The cells were then resuspended in Dulbecco's Modified Eagle Medium (DMEM, Gibco) supplemented with 10% fetal bovine serum (FBS, Gibco) and penicillin (100 U/ml) and streptomycin (100 μg/ml). This mixture was designated as complete growth medium (CGM) and was incubated at 37°C in a humidified atmosphere containing 5% CO_2_ until further experimentation.

### 2.3 Limiting dilution clonogenic assay for lymphocytes

To evaluate lymphocyte radiosensitivity, a limiting dilution clonogenic assay adapted for lymphocyte cultures was performed.

PBMCs were diluted to a concentration of 10^5 cells/mL in DMEM, supplemented as previously described, together with 10% fetal bovine serum (Gibco), 1% heat-inactivated human serum (obtained from a single normal individual), 1% phytohemagglutinin (Gibco), and 10 U/mL of human recombinant interleukin-2 (Immune Teb Padideh, Iran). Cells were seeded into 96-well Terasaki plates at densities of 17 cells/well for control (0 Gy) and 20 cells/well for irradiated samples (2 Gy). The slightly higher seeding density in irradiated samples was used to compensate for expected radiation-induced cell loss.

Plates were incubated for 10–14 days, after which wells were examined using an inverted phase-contrast microscope to determine the presence or absence of colony growth. Colony-forming efficiency and survival parameters were calculated according to previously described methods [19].

In brief, colony-forming efficiencies (CFE) were calculated using the formula: CFE = ln(n/t)/c, where n represents the number of wells without colonies, t denotes the total number of seeded wells, and c indicates the number of cells seeded per well.

### 2.4 Analysis of DNA double strand breaks and repair kinetics

DNA double strand breaks and repair kinetics were assessed through the measurement of histone H2A.X phosphorylation (γH2AX) in PBMCs following irradiation.

Irradiated cells were divided into three groups: 30 min, 3 h and 24 h after irradiation and assessed according to the standard protocol of the H2AX phosphorylation detection kit (Millipore, USA). First, the cells were fixed with a formaldehyde/methanol solution and incubated on ice for 20 min. After washing with PBS, the cells were permeabilized using 1× permeabilization solution. Subsequently, 3.5 μL of FITC-conjugated anti-phosphorylated H2AX antibody (Ser139) was added to the cell pellet and incubated on ice for another 20 min.

After staining, cells were washed with PBS/saponin solution and analyzed using a two-color FACSCalibur flow cytometer (Becton Dickinson, USA). Flow cytometry data were processed and analyzed using FlowJo software (version V10, FlowJo, USA).

The γH2AX signal intensity was used as an indicator of DNA double-strand break formation and repair dynamics after radiation exposure.

### 2.5 In vitro X-ray irradiation

The isolated PBMCs were adjusted to a final density of 1 × 10^6^ cells/ml and incubated at 37°C in a 5% CO2 atmosphere. Irradiation was performed using a 6-MV linear accelerator (Elekta Compact) at a dose rate of 2 Gy/min, 10 × 10 cm^2^ field size, source-to-surface distance (SSD) of 100 cm and 2.5 cm build-up with Plexiglas and 180° gantry angle, delivering a total dose of 2 Gy. Samples were maintained under controlled laboratory conditions during irradiation. Non-irradiated samples served as controls and were handled under identical experimental conditions.

### 2.6 Collection of breast cancer tissue samples

Breast tumor specimens were collected during surgery under sterile conditions. Frozen section analysis was performed intraoperatively to confirm the pathological nature of the tissue.

Following surgical excision, tissue samples were immediately placed in sterile containers containing complete growth medium (CGM) and transported to the central laboratory within 1 h to preserve cell viability.

### 2.7 Isolation and culture of breast cancer cells and breast cancer stem cells

Tumor tissues were mechanically minced into small fragments and subjected to enzymatic digestion using: 0.05% type II collagenase (Invitrogen, Germany) and 0.05% trypsin (Sigma). Digestion was carried out at 37°C for 30 min in a sterile incubator.

The resulting suspension was centrifuged at 1200 rpm for 5 min and filtered through a 70 μm cell strainer (SPL) to obtain a single-cell suspension. Cells were cultured in DMEM/F12 medium (Gibco) supplemented with 10% fetal bovine serum (FBS) and 1% penicillin-streptomycin (100 U/ml and 100 μg/ml, respectively). The single-cell suspensions derived from BC tissues were divided into two fractions: 1. monolayer culture of breast cancer cells and 2. enrichment of breast cancer stem cells (BCSCs).

### 2.8 Enrichment and identification of breast cancer stem cells

BCSCs were enriched using a sphere formation assay under serum-free conditions. Cells were cultured in low-attachment plates containing serum-free DMEM/F12 supplemented with 20 μg/L epidermal growth factor (EGF), 20 μg/L basic fibroblast growth factor (bFGF), and 2% B27 supplement. Sphere formation was monitored during culture, and fresh medium was added three days after initiation.

To characterize the stem-like population, cells were analyzed using flow cytometry (FACSCalibur, Becton Dickinson, USA) for CD44 and CD24 expression, which are widely used markers for breast cancer stem-like cells.

### 2.9 Clonogenic survival assay of breast cancer and breast cancer stem cells

Breast cancer cells and enriched BCSCs were plated in 6-well plates at densities of: 1000 cells/well (control group) and 1500 cells/well (irradiated group). The increased seeding density in the irradiated group was used to compensate for radiation-induced loss of clonogenic cells. Twenty-four hours after seeding Cells were irradiated with 2 Gy and followed by 2–3 weeks incubation to allow colony formation.

Colonies were then fixed with methanol/acetic acid (3:1) and stained with Giemsa. Colonies containing more than 50 cells were counted using ImageJ software (version 1.41o, Java 1.6.0_10, Wayne Rasband, Bethesda, MD, USA).

Each experiment was performed in triplicate, and colony counts were used to calculate clonogenic survival parameters.

The survival fraction at 2 Gy (SF2) was calculated as:

SF2 = (colonies counted after irradiation) / (number of cells seeded) × plating efficiency (PE)

Where plating efficiency (PE) represents the ratio of colonies formed to cells seeded in the control group.

### 2.10 Statistical analysis

Statistical analyses were performed using GraphPad Prism version 8. All experiments were conducted in three independent replicates, and results are presented as mean ± standard deviation (SD). Data distribution was evaluated using the Kolmogorov–Smirnov test. Correlations between radiosensitivity parameters of different cell populations were assessed using the Pearson correlation coefficient. All statistical tests were two-tailed, and a p-value <0.05 was considered statistically significant. Graphical representations were generated using GraphPad Prism and Microsoft Excel (version 2019).

## 3 Results

Immunohistochemical analysis of frozen breast cancer tissue obtained during biopsy was used to evaluate the pathological features of the enrolled patients. The expression status of HER2 and hormone receptors, including estrogen and progesterone receptors, is summarized in Table 1. In addition, the relationship between lymphocyte SF2 and γH2AX-related parameters was examined.

**Table 1:** Results of Immunohistochemistry and pathological information of BC patients.

	age	grade	ER	PR	HER2	Ki67
C1	35	3	-	-	-	70%
C4	47	2	+	-	-	15%
C5	52	2	+	-	-	50%
C6	56	2	+	+	+	10%
C7	52	2	+	+	-	20%
C8	46	3	-	-	+	45%
C10	47	2	-	+	-	10%
C11	42	3	+	+	+	15%
C12	63	2	-	+	-	20%
C13	52	2	+	-	-	10%
C14	55	2	+	+	+	30%
C15	38	2	-	-	-	25%
C16	38	2	-	+	+	20%
C17	51	3	+	+	-	50%
C18	44	2	+	+	+	30%
C19	48	2	+	+	-	20%
C20	58	2	-	-	+	10%
C21	70	-	-	-	-	5%
C22	66	2	+	+	-	10%
C23	54	2	+	-	-	20%

ER=Estrogen Receptor, PR=Progesterone Receptor, HER2= Human epidermal growth factor receptor-2.

**Fig. 1:**
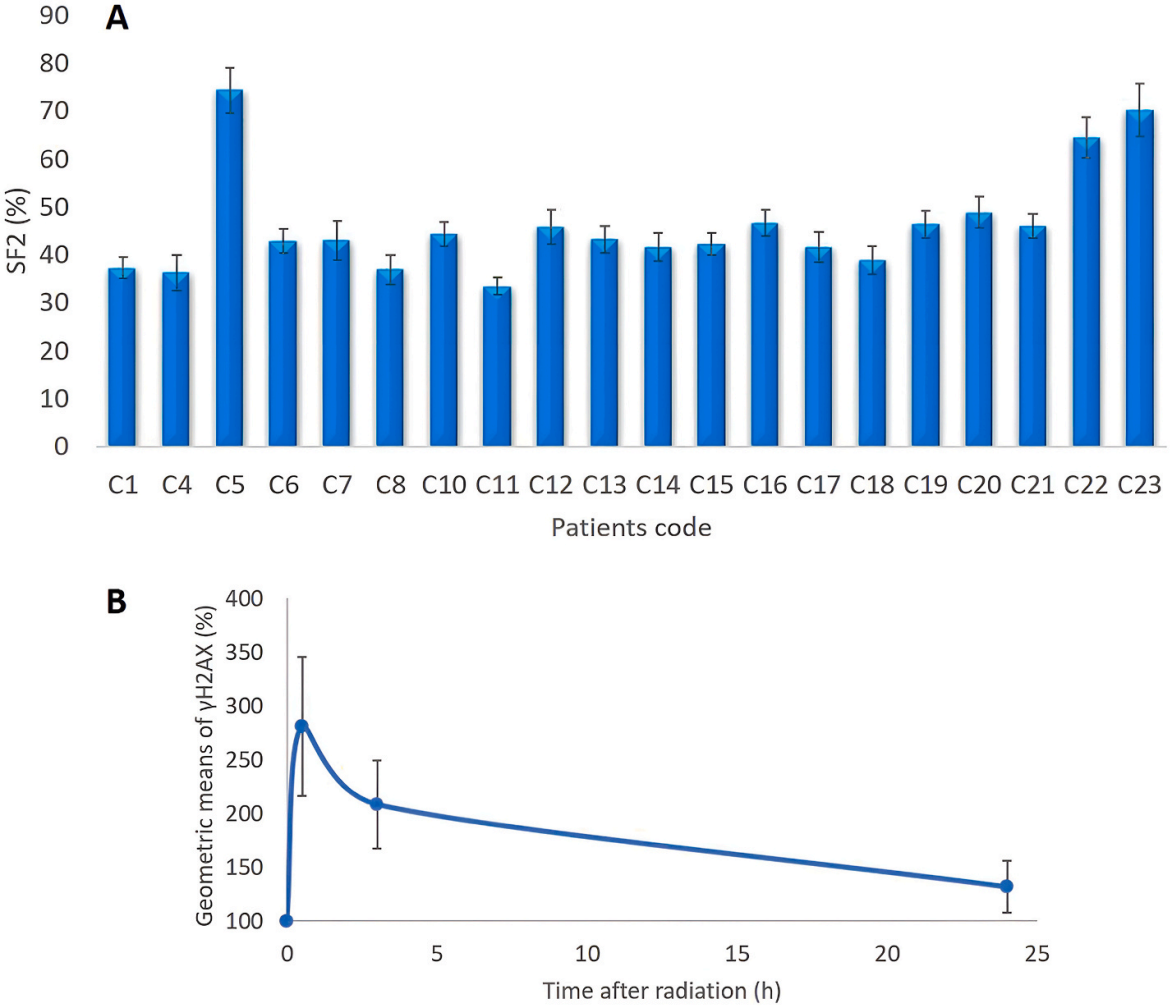
(A) Survival fraction in 2Gy of lymphocytes for each patient. (B) Mean of the normalized geometric means for patient lymphocytes in 24h after irradiation with 2Gy.

SF2 values were calculated for lymphocytes from all participants, as shown in Fig. 1A. Among the patients, C5 showed the lowest lymphocyte radiosensitivity, whereas C11 showed the highest. The mean SF2 across the cohort was 46.213 ± 11.

To assess DNA double-strand break formation and repair in peripheral blood lymphocytes after exposure to 2 Gy, the γH2AX assay was performed. Flow cytometry was used to measure the geometric mean at 0.5, 3, and 24 h after irradiation for each patient. The changes observed over this 24-h period are shown in Fig. 1B.

The geometric mean at 0.5 h likely reflected initial DNA damage, whereas the values at 3 and 24 h were used to estimate repair capacity. Pearson's correlation analysis was performed to evaluate the association between these parameters and lymphocyte SF2, as summarized in Table 2. The geometric mean at 30 min showed a significant correlation with the SF2 of lymphocytes.

**Table 2:** Survival fraction (SF2) and normalized geometric means for all patients at 3 time after radiation and correlation coefficient between them.

Patients code	SF2	Normalized Geometric Means at 0.5 h after radiation	Normalized Geometric Means at 3 h after radiation	Normalized Geometric Means at 24 h after radiation
C1	37.25	151.5525	256.2839	114.4899
C4	36.22	183.023	284.0013	157.7785
C5	74.29	337.9092	287.4575	191.2472
C6	42.91	373.5051	253.9098	226.3109
C7	42.99	302.748	209.5948	147.2287
C8	36.87	149.1265	163.3291	161.9088
C10	44.32	342.6926	91.31749	102.0446
C11	33.45	193.75	203.5714	172.3214
C12	45.8	297.4747	162.1212	108.0808
C13	43.2	262.8664	238.4365	152.1173
C14	41.6	184.3818	125.3796	85.90022
C15	42.3	159	145.5	91
C16	46.6	487.3418	256.3291	155.6962
C17	41.61	355.625	285.625	198.75
C18	38.81	242.0253	214.6835	83.03797
C19	46.38	547.7528	439.8876	193.8202
C20	48.9	117.8478	92.38845	84.7769
C21	46.11	201.2245	135.102	84.4898
C22	64.52	169.2153	134.6076	53.31992
C23	70.13	562.1622	189.7297	72.43243

		SF2-Lymph vs. Geometric Means in 0.5 h after radiation	SF2-Lymph vs. Geometric Means in 3 h after radiation	SF2-Lymph vs. Geometric Means in 24 h after radiation
Pearson's correlation coefficient	r	0.3921	−0.01746	−0.2047
	P-value	0.0436^a^	0.4709	0.1933

a Significant difference.

Using this assay, two additional metrics can be derived: the remaining damage index at 3 h and at 24 h after radiation exposure. The geometric mean at 30 min demonstrated a significant correlation with the surviving fraction at 2 Gy (SF2) of lymphocytes.

Residual double-strand breaks (DSBs) indicate the extent of unrepaired damage, assessed at subsequent time points using γH2AX. This quantification is calculated as follows:

% Residual DSBs = [(Geometric mean of γH2AX (3 or 24 h) − Geometric mean of γH2AX (control)) / (Geometric mean of γH2AX (30 min) − Geometric mean of γH2AX (control))] × 100.

**Fig. 2:**
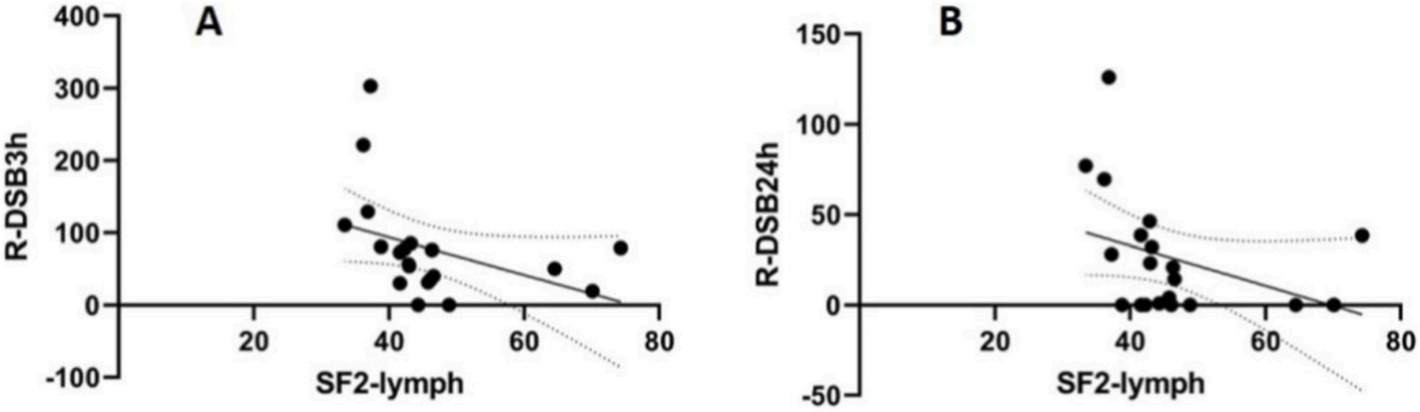
Scatter plots with correlation between the SF2 of lymphocytes and residual DSBs at 3 and 24 h after 2Gy irradiation in breast cancer patients.

As shown in Fig. 2A and B, SF2 showed a significant inverse correlation with residual DSBs at 3 h post-irradiation (r = −0.3923, P = 0.0435). Residual DSBs at 24 h were also inversely associated with SF2, although this did not reach statistical significance (r = −0.3629, P = 0.0579).

Breast cancer stem cells (BCSCs) were then characterized. During culture in low-attachment plates, the cells initially appeared round. Small spheroids became visible within the first three days and increased in size with daily medium changes. Sphere formation was clearly evident after 72 h.

**Fig. 3:**
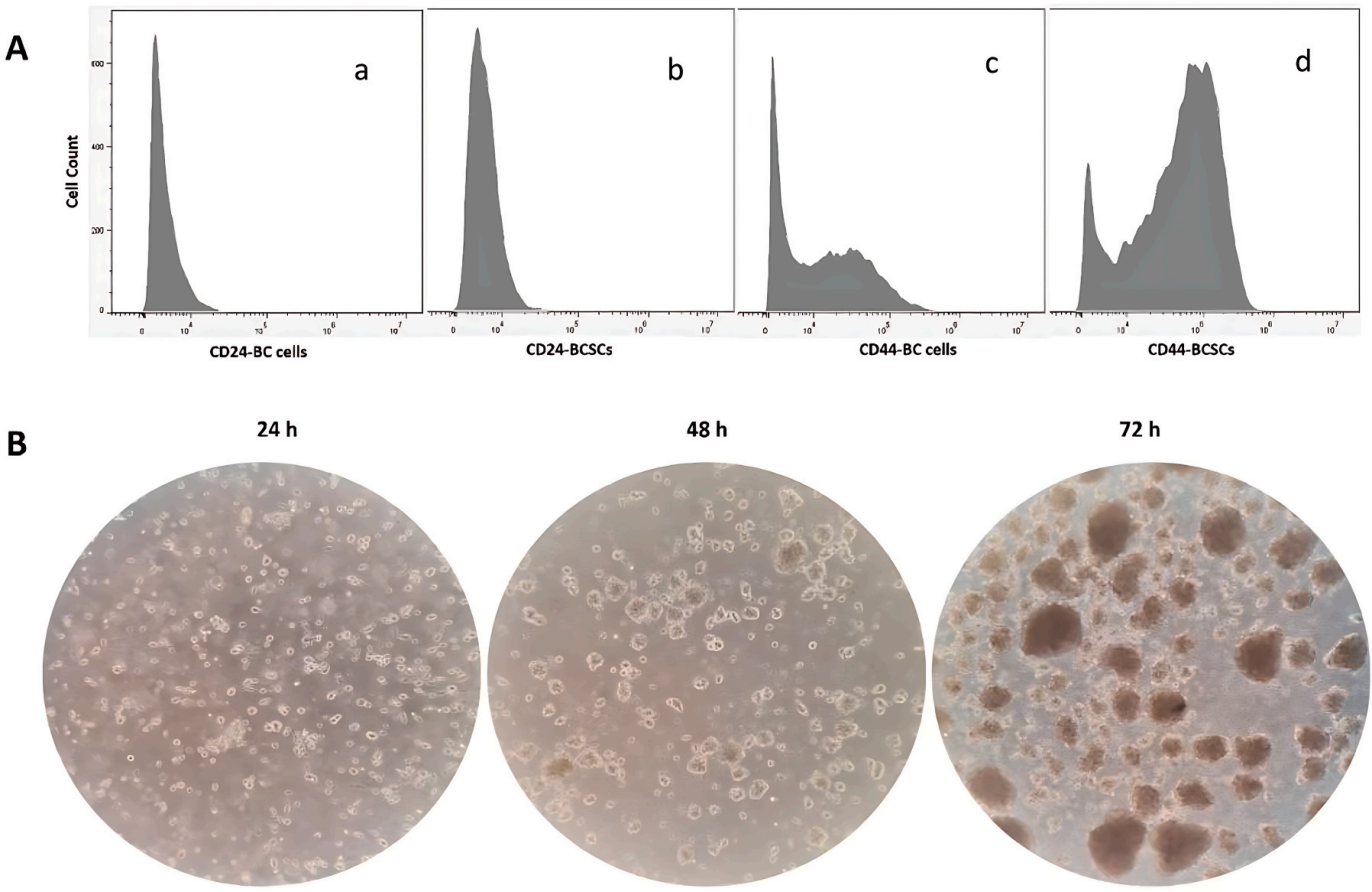
(A) Histogram of CD markers of breast cancer (BC) cells and breast cancer stem cells (BCSC)-derived BC cells with flow cytometry. a: CD24 in BC cells, b: CD24 in BCSC, c: CD44 in BC cells and d: CD44 in BCSCs. (B) Spheroid formation of BC cells after 24, 48 and 72h culture in ultra-attachment plates.

Flow cytometry was used to assess the CD44+/CD24-/low population before and after isolation. Before enrichment, this population accounted for 24.5 ± 2.6% of the total cell suspension. After enrichment, this population increased to 65.04 ± 9.74%. Fig. 3A representative flow cytometry profiles from one patient breast cancer cells and the isolated BCSCs, and Fig. 3B shows spheroid formation in low-attachment culture.

### 3.1 Correlation between SF2 in lymphocytes, breast cancer cells, and BCSCs

After culture and clonogenic assessment, no colonies were obtained from samples of five patients, and these cases were excluded from the final analysis. Colony counts from the remaining fifteen patients were used to calculate SF2 in the different cell populations.

**Fig. 4:**
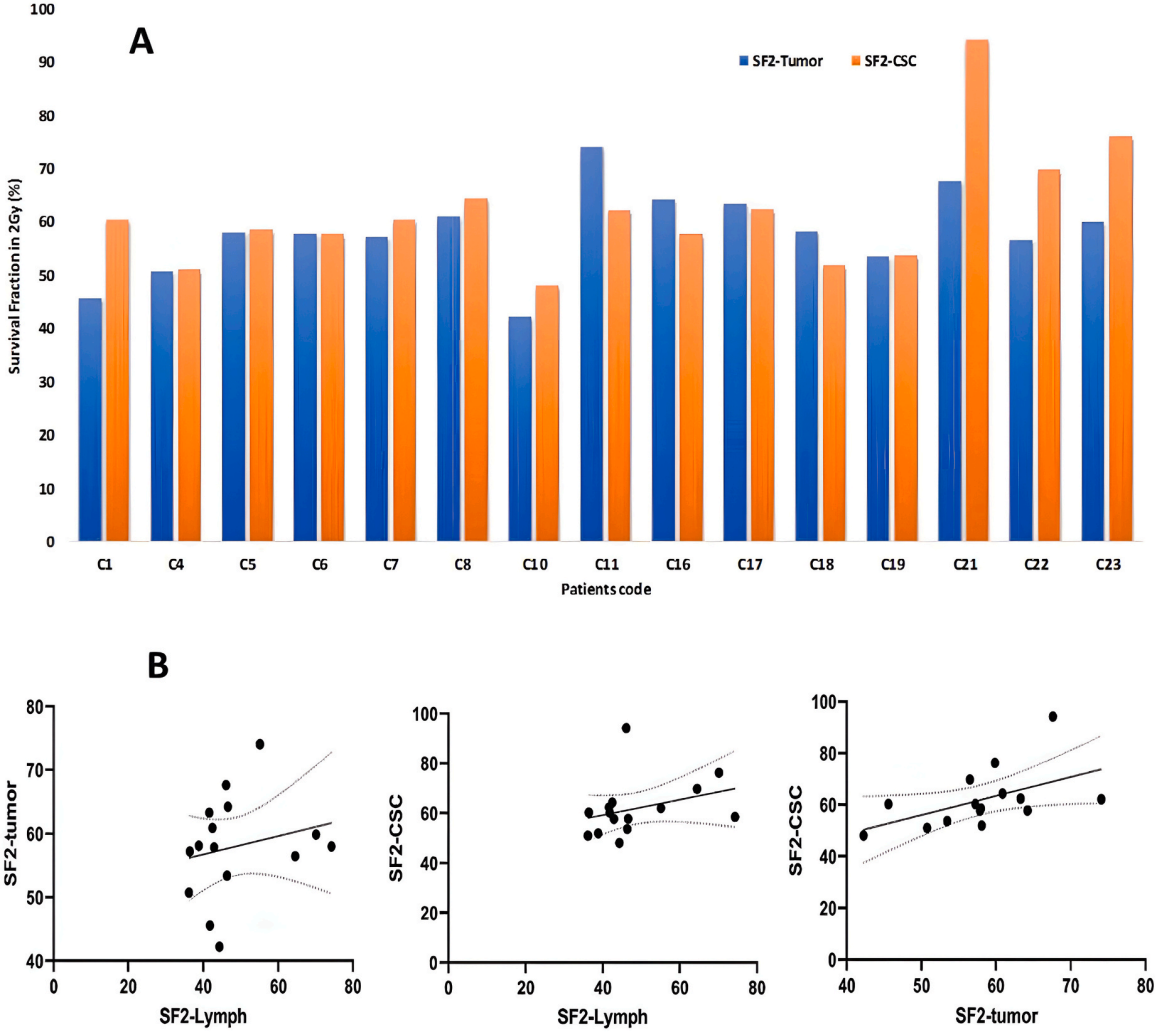
(A) The survival fraction in 2Gy of tumor cells and BC stem cells. (B) Scatter plots with correlation between the survival fraction of lymphocytes, BC cells (tumor) and breast cancer stem cells (CSC).

The plating efficiency of breast cancer cells ranged from 4.4% to 16%, with a mean of 7.5%. In BCSCs, the mean plating efficiency was 5.76%, with a range of 3.15% to 8.53%. Fig. 4 presents the SF2 values for breast cancer cells and BCSCs from the 15 remaining patients.

Among breast cancer cells, C11 showed the lowest radiation sensitivity, whereas C10 showed the highest. The mean radiosensitivity of breast cancer cells was 57.98 ± 9.06% (Fig. 4A). In BCSCs, C21 showed the lowest radiation sensitivity and C10 the highest, with a mean value of 61.9 ± 12.46% (Fig. 4A). Fig. 4B shows the correlation between SF2 in lymphocytes, breast cancer cells, and breast cancer stem cells.

## 4 Discussion

The present study investigated the radiosensitivity using clonogenic assays in different cell populations and γH2AX analysis in lymphocytes. The results of the γH2AX assay indicated that residual double-strand breaks (DSBs) represent a more reliable closely associated indicator of lymphocytes radiosensitivity than the initial damage reported in prior studies [[34], [35], [36]]. This finding suggests that the γH2AX assay may provide a more precise assessment of radiation sensitivity in normal cells and may provide complementary information on DNA repair capacity than clonogenic survival assays. Notably, no significant correlation was observed between the radiosensitivity of lymphocytes and that of tumor cells or tumor stem cells. As expected, BCSCs exhibited reduced radiosensitivity, likely due to their distinct signaling pathways.

The survival fraction at 2 Gy (SF2) for lymphocytes showed substantial inter-individual variability, ranging from 33.45% to 74.29%, with a mean value of 46.21 ± 10.95%. This variability is consistent with findings from other studies, which report marked differences in radiation sensitivity across patient populations [[37], [38], [39], [40]]. The considerable variability—exceeding 40%—in radiation sensitivity among patients likely resulted in a lack of statistical correlation, thereby limiting the clinical applicability of these observations [41].

**Fig. 5:**
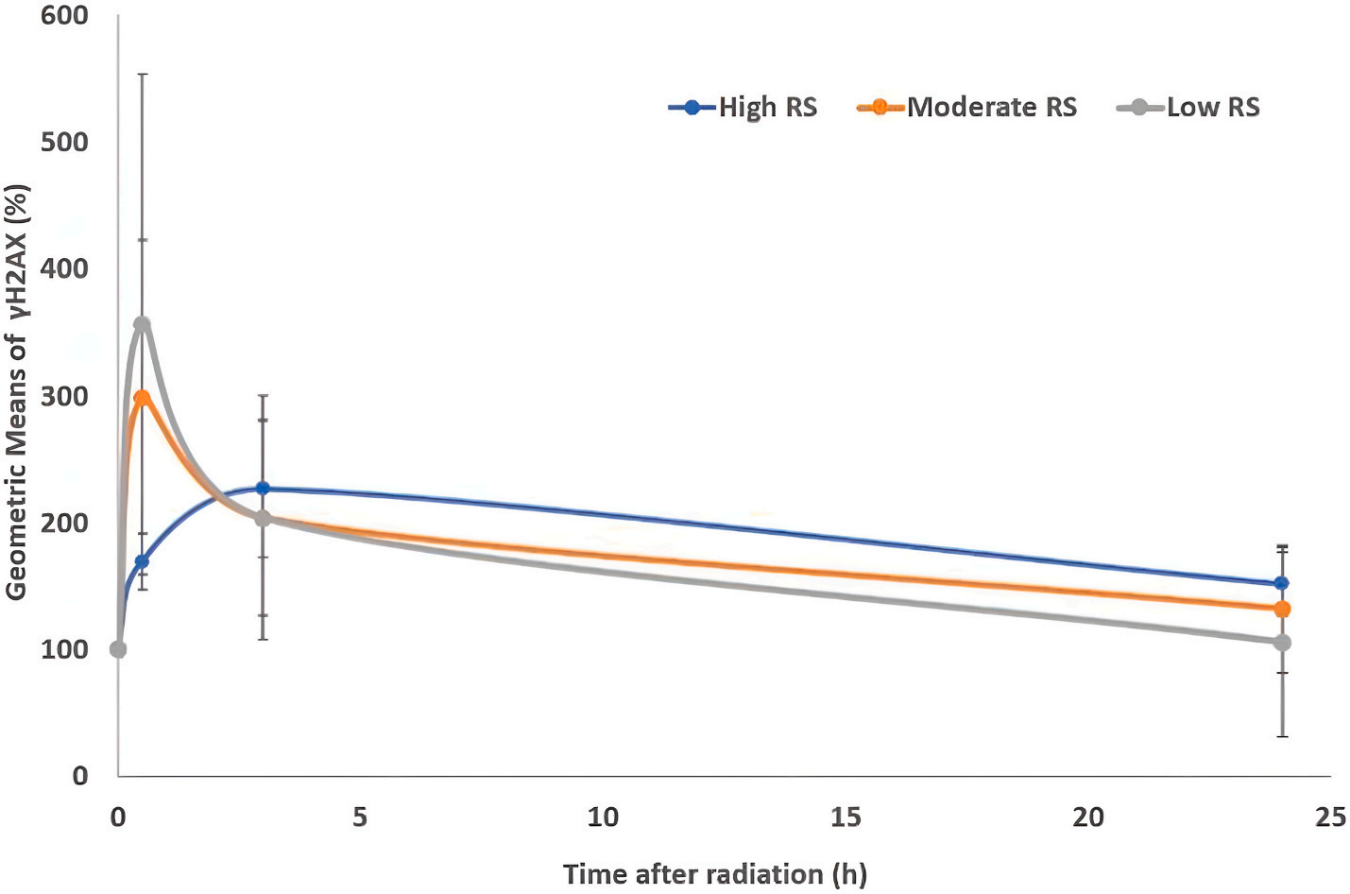
The geometric means of γH2AX at 3 times after radiation in different radiosensitive (RS) groups: (blue line) high radiosensitive patients, (orange line) moderate radiosensitive patients and (gray line) low radiosensitive patients.

In this study, we evaluated the radiosensitivity of peripheral blood lymphocytes in patients with breast cancer using the γH2AX assay. We compared the outcomes obtained at three time points after irradiation with those derived from colony-forming assays. Consistent with previous studies [35,42], we observed peak fluorescence intensity at 30 min post-irradiation, followed by a gradual decline over the subsequent 24 h. However, in four patients displaying the lowest SF2 values, the highest fluorescence intensity was detected at 3 h post-irradiation, which may indicate deficiencies in DNA repair mechanisms. To further interpret these findings, patients were classified into three categories based on lymphocyte SF2: radiation-sensitive, radiation-resistant, and moderately sensitive. Differences in γH2AX fluorescence intensity suggested the presence of defects in DNA repair mechanisms among the radiation-sensitive subgroup (Fig. 5).

Our findings suggest that residual DNA damage may better reflect cellular radiosensitivity than the initial damage level. In the present cohort, residual DSBs measured several hours after irradiation showed a stronger association with lymphocyte SF2 than the early damage signal. Previous investigations have similarly emphasized the importance of DNA repair efficiency as a determinant of radiosensitivity [43,44]. In particular, Bourton et al. reported that patients with higher radiation sensitivity exhibited reduced DNA repair capacity compared with those with minimal adverse effects [45]. Djuzenova et al. observed a greater extent of residual damage in cancer patients than in healthy individuals [46], while Vasireddy et al. reported increased γH2AX foci in highly sensitive patients following radiation exposure [47]. In addition, Yin et al. and Kroeber et al. emphasized the relationship between unrepaired damage and treatment efficacy [48,49].

The present study also examined the association between initial damage and residual double-strand breaks (DSBs), demonstrating that residual damage was more strongly correlated with the surviving fraction at 2 Gy (SF2) than initial damage. Similar findings have been reported in different studies, highlighting that cell type substantially influences these outcomes [34]. Reun et al. reported a strong correlation between residual damage assessed 24 h after exposure and SF2; however, the variability in residual damage complicated the discrimination of individuals with moderate sensitivity [36]. In conclusion, the γH2AX assay proved to be an effective method for evaluating radiosensitivity in BC patients, providing important insights into inter-individual differences in radiation response and potential relevance for future radiobiological studies. It is important to note that peripheral blood lymphocytes are among the most radiosensitive cells in the human body and are readily accessible, which is why this cell type has been employed in many studies to assess intrinsic radiosensitivity. In contrast, sampling other normal cells usually requires invasive procedures and tissue biopsies; therefore, using lymphocytes represents a less invasive and more practical approach for introducing a new method.

However, it should be emphasize that radiosensitivity varies among healthy cells of different tissues and could influence the study outcomes. Consequently, a comprehensive evaluation of the radiosensitivity of healthy tissues surrounding the tumor within the radiotherapy field is recommended for further investigations.

A wide range of SF2 values has been observed in both tumor cells and tumor stem cells among BC patients, consistent with prior reports [[50], [51], [52], [53], [54]]. Using “mean ± one standard deviation” values, which account for approximately 70% of data in a normal distribution, it is estimated that approximately 70–80% of individuals exhibit moderate radiation sensitivity, whereas only 20–30% are categorized as either radiation-sensitive or radiation-resistant.

Cancer stem cells possess distinct properties that confer resistance to multiple treatment modalities, thereby substantially contributing to tumor recurrence. Evidence indicates that the radiosensitivity of breast cancer stem cells (BCSCs) is slightly lower than that of conventional BC cells, with an SF2 of 62% for BCSCs compared with 58% for BC cells. Numerous studies have assessed survival of CSCs across diverse cancer types. For instance, Ke et al. (2020) reported an SF2 of 61% for laryngeal cancer stem cells, whereas non-stem cells showed a markedly lower SF2 of 18% [55]. Similarly, Wang et al. found elevated survival rates in esophageal cancer stem cells, with SF2 values of 81% and 87% compared with 69% and 80% for their parental cells [56]. Bertrand et al. (2014) observed an SF2 of 82% for cervical cancer stem cells versus 56% for parental cells even after carbon ion irradiation [57]. Blazek et al. investigated glioblastoma and reported increased survival in stem-like cells, with SF2 values of 60% for CSCs compared with 58% for glioblastoma cells [58]. Zhong et al. reported that kidney carcinoma stem cells had an SF2 of 74%, whereas tumor cells had an SF2 of 65% [59].

Mihatsch et al. conducted a study involving two breast cancer cell lines, demonstrating that the A549 cell line exhibited a substantially higher survival rate than the tumor cells (53% versus 40%). By contrast, this difference was not statistically significant in the SK-BR-3 cell line. Radiation resistance in CSCs is hypothesized to be associated with enhanced DNA repair capacity and effective apoptotic responses [60]. In a study by Chen et al. (2018), results for A549 appeared contradictory, indicating that CSCs exhibited greater radiation sensitivity than their parental cells, potentially due to differences in culture conditions [61]. Overall, although this research is consistent with many studies suggesting a general trend of radiation resistance in CSCs, the magnitude of this resistance appears to vary across cell and experimental conditions. A comprehensive understanding of these dynamics is essential for the development of targeted therapies aimed at mitigating CSC-mediated treatment resistance and improving patient outcomes.

An additional aim of this study was to investigate whether the radiosensitivity of lymphocytes could reflect the radiosensitivity of tumor cells in the same patient. Pearson correlation analysis did not reveal a significant relationship between these parameters (see Table 3). Despite advances in radiosensitivity assessment, studies linking the radiosensitivity of normal and tumor cells remain limited. Most existing studies focus on the effects of radiotherapy (RT) on normal tissues, which may require modification or discontinuation of treatment due to radiation-induced damage. Conflicting results have been reported from studies comparing colony-forming capacity between normal and tumor cells: some report a significant correlation between the SF2 of normal cells and tumor radiosensitivity, whereas others do not [22,50,[62], [63], [64]]. A shared genetic background has been proposed as one factor influencing the relationship between radiation sensitivity in healthy and tumor cells. The presence of multiple mutations in tumor cells may disrupt this relationship, leading to inconsistent radiosensitivity even among different normal cell types within the same individual [65].

**Table 3:** The correlation between breast cancer cells and breast cancer stem cells with parameters of γH2AX assay.

		Normalized Geometric Means in 0.5 h after radiation	Normalized Geometric Means in 3 h after radiation	Normalized Geometric Means in 24 h after radiation	R-DSB3h	R-DSB24h
Tumor cells	r	−0.004395	−0.05089	0.1648	−0.2967	0.2273
	P value	0.4938	0.4285	0.2786	0.1415	0.2076
BCSCs	r	−0.1138	−0.3825	−0.4084	−0.2288	−0.1834
	P value	0.3432	0.0797	0.0654	0.2060	0.2564

## 5 Limitations

Several limitations of this study should be acknowledged. It should be considered a preliminary exploratory analysis with a relatively small sample size and a number of samples could not be included in the final clonogenic analysis because colony formation was insufficient. In addition, the biological heterogeneity of breast cancer subtypes may influence radiosensitivity and could contribute to the variability observed in the results. Larger studies that incorporate molecular characterization of tumors may therefore help clarify the relationship between radiosensitivity and tumor biology.

Overall, the findings of this study highlight the complexity of predicting individual radiation response. Although assays such as SF2 and γH2AX provide useful biological information, none of them alone appears sufficient to fully characterize intrinsic radiosensitivity. Integrating multiple biological markers and analytical approaches with simultaneous cell-cycle assessment may therefore be necessary to improve the predictive value of radiosensitivity assessments. So, available clinical outcome data, including treatment response or toxicity follow-up, could decrease uncertainty of predictive value of SF2 and γH2AX-related parameters. Finally, the findings should not be overgeneralized to clinical decision-making, and further validation in larger cohorts is required.

## 6 Conclusion

This preliminary study evaluated the radiosensitivity of peripheral blood lymphocytes, breast cancer cells, and breast cancer stem cells using clonogenic survival assays and γH2AX-based measurements of DNA damage. Considerable inter-individual variability in radiosensitivity was observed among patients. Residual DNA damage measured by the γH2AX assay showed a closer association with lymphocyte survival than the initial damage signal, suggesting that DNA repair capacity may contribute to cellular radiation response. However, no significant correlation was detected between the radiosensitivity of lymphocytes and that of tumor cells or breast cancer stem cells. These findings suggest that currently available assays, including SF2 and γH2AX-based measurements, provide only partial information on intrinsic radiosensitivity. In the absence of clinical outcome data, the predictive relevance of these parameters for treatment response or normal tissue toxicity remains uncertain. Overall, γH2AX should be considered a complementary assay rather than a substitute for clonogenic analysis. Further studies in larger cohorts, with molecular tumor characterization and clinical follow-up, are needed to clarify the potential value of these markers in future individualized radiotherapy research.

## Ethical approval

The study was approved by the Mashhad University of Medical Sciences Ethics Committee (IR.MUMS.MEDICAL.REC.1398.533).

Informed Consent: all participants fill out the informed consent form before sampling.

Registry and the Registration No: N/A.

Animal Studies: N/A.

## Declaration of used to AI technology

During the preparation of this work, the authors the authors used ChatGPT and NotebookLM to improve language quality, check grammar, and enhance the structure and clarity of the manuscript. After using this tool, the authors reviewed and edited the content as needed and take full responsibility for the content of the published article.

## Funding

The authors would like to thank the “Office of the Vice President for Research of Mashhad University of Medical Sciences, Mashhad, Iran” and the “Iran National Science Foundation” for funding this work.

## CRediT authorship contribution statement

**Mohammad Taghi Bahreyni Toossi:** Project administration. **Hosein Azimian:** Methodology, Supervision. **Roham Salek:** Validation. **Seyed Abbas Tabatabaei:** Conceptualization, Formal analysis. **Mohammad Naser Forghani:** Data curation. **Elham Dolat:** Data curation, Formal analysis, Investigation, Writing – original draft.

## Declaration of competing interest

The authors declare that they have no known competing financial interests or personal relationships that could have appeared to influence the work reported in this paper.

## Data Availability

Data will be made available on request.
